# Utilization of High Performance Liquid Chromatography Coupled to Tandem Mass Spectrometry for Characterization of 8-*O*-methylbostrycoidin Production by Species of the Fungus *Fusarium*

**DOI:** 10.3390/jof3030043

**Published:** 2017-07-25

**Authors:** Mark Busman

**Affiliations:** Mycotoxin Prevention and Applied Microbiology Research Unit, National Center for Agricultural Utilization Research, Agricultural Research Service, United States Department of Agriculture, Peoria, IL 61604, USA; Mark.Busman@ars.usda.gov; Tel.: +1-309-681-6241

**Keywords:** maize, liquid chromatography-mass spectrometry, 8-*O*-methylbostrycoidin, pigment

## Abstract

The pigment 8-*O*-methylbostrycoidin is a polyketide metabolite produced by multiple species of the fungus *Fusarium* that infects plant crops, including maize. A technique was developed for the analysis of 8-*O*-methylbostrycoidin by high performance liquid chromatography coupled to electrospray ionization tandem mass spectrometry. The quantitative nature of the LC-MS/MS experiment was demonstrated over a range of concentrations in maize. Limits of detection for the method (10 ng/g from 8-*O*-methylbostrycoidin spiked into ground maize) were shown, and susceptibility of the method to matrix effects from maize was also evaluated. The method was applied to evaluate the ability of the maize pathogen *Fusarium verticillioides* to produce 8-*O*-methylbostrycoidin in developing maize ears grown in an agricultural field.

## 1. Introduction

**Figure 1:**
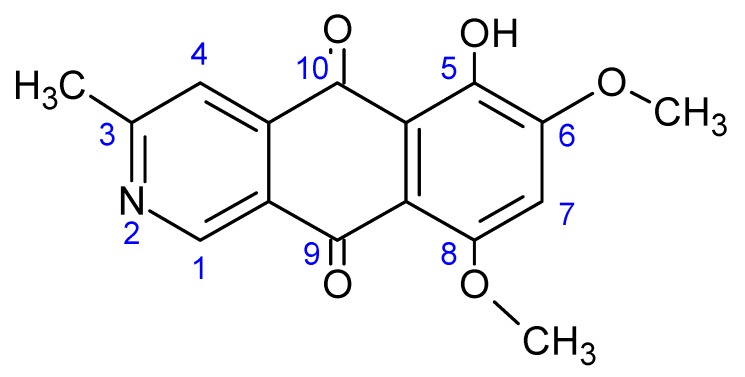
Structure of 8-*O*-methylbostrycoidin.

The pigment, 8-*O*-methylbostrycoidin (Figure 1), is an azaanthraquinone secondary metabolite that is widely produced by species of the filamentous fungus *Fusarium*. A complete structural characterization was accomplished by Steyn [1]. Total synthesis of the compound was demonstrated by Cameron [2,3]. The compound was first observed from *Fusarium* isolates taken from maize [1] and citrus [4]. Deng has recently noted production from a marine *Aspergillus* isolate [5].

Early studies described the isolation of 8-*O*-methylbostrycoidin and showed its cytotoxicity against several mammalian cell lines, including rat hepatoma, Madin Darby canine kidney and Chinese hamster ovary [6]. Later, Hashimoto again observed the low cytotoxicity against the rat hepatoma cell line, but observed a strong ability to inhibit hepatic glucose production [7]. More recently, 8-*O*-methylbostrycoidin has been evaluated for cytotoxicity to a number of human cancer cell lines and has been shown to strongly inhibit α-acetylcholine esterase [5].

It is thought that 8-*O*-methylbostrycoidin biosynthesis involves a polyketide synthase that catalyzes the synthesis of a heptaketide, the first intermediate in 8-*O*-methylbostrycoidin biosynthesis. It has been suggested that a common polyketide synthase is responsible for the initial stages in the synthesis of several anthraquinones produced by *Fusaria* [8,9,10].

Chemical characterization of 8-*O*-methylbostrycoidin frequently is based upon its deep red color. For example, 8-*O*-methylbostrycoidin production in fungi grown on culture plates can be monitored visually, based on the color of the substrate. Levels of 8-*O*-methylbostrycoidin have also been monitored by absorbance at 510–520 nm either in HPLC detection or in spectrophotometric analysis of *Fusarium* extracts. Such UV-VIS based detection of related compounds has been implicated as an interfering actor in the detection of another Fusarium metabolite, zearalanone [11].

Here, we demonstrate a convenient technique for the analysis of 8-*O*-methylbostrycoidin by high performance liquid chromatography coupled to electrospray ionization tandem mass spectrometry. The technique is applied to the screening of *Fusarium* species for 8-*O*-methylbostrycoidin production on cracked maize substrate and to the evaluation of field production by *F. verticillioides* in maize.

## 2. Materials and Methods

### 2.1. Materials

Unless otherwise noted, all solvents were of HPLC grade from Sigma Chemical Co. (St. Louis, MO, USA). Water was from a Millipore (Billerica, MA, USA) water purification system. Purified 8-*O*-methylbostrycoidin was obtained from cultures of *F. verticillioides* as previously described [6]. Mature cultures of *F. verticillioides* MRC-826 on cracked maize were extracted with 60% aqueous methanol and purified on with successive Amberlite XAD-2 (Sigma, St. Louis, MO, USA) and normal phase (silica gel) liquid chromatographies. Eluted 8-*O*-methylbostrycoidin was monitored by ESI-MS. Purified 8-*O*-methylbostrycoidin was evaluated by ^1^H and ^13^C NMR. A stock solution of 1 mg/mL 8-*O*-methylbostrycoidin in acetonitrile was used for constructing calibration solutions and for spike solutions.

### 2.2. Fungal Strains and Preparation of Maize Materia

Two strains of *F. verticillioides* isolates were obtained from field-grown maize kernels as previously described [12]. Briefly, maize kernels were surface sterilized by soaking in a 10% chlorine bleach solution for 1 min. After rinsing three times in sterile water, the kernels were placed on Nash agar medium [12] and incubated for 3–5 days in the dark at room temperature. Single-spore derived isolates of the resulting fungal growth were obtained as described previously [13]. The resulting colonies were identified as *F. verticillioides* first by morphology in comparison with morphological descriptions of the fungus [13] and then by DNA sequence analysis of the gene encoding the translation elongation factor 1-α and comparison of the resulting sequence data to the Fusarium ID Database [14]. Cracked maize kernel cultures of *Fusarium* were prepared as previously described [15].

### 2.3. Extraction of 8-O-methylbostrycoidin from Maize

Twenty grams of cracked or ground maize were accurately weighed and extracted with 50 mL of acetonitrile for 2 h on a Gyratory Model G2 Shaker (New Brunswick Scientific, Edison, NJ, USA). Slurries of sample with solvent were centrifuged for 5 min at ~1500× *g*. One milliliter aliquots of the centrifugate were decanted by pipette into sample vials for LC-MS analysis.

### 2.4. ESI-MS and MS/MS

Unless otherwise noted, all experiments were conducted utilizing a ThermoFinnigan LCQ-DECA (Thermo Scientific, San Jose, CA, USA) ion trap mass spectrometer equipped with an electrospray ionization source. Flow injection experiments were accomplished with by injecting 10 µL plugs of analyte solution into a 300 µL/min flow of 1/1 methanol-water with 0.3% acetic acid. Injection of the analyte plugs was done by use of a Rheodyne Model 7125 injector (IDEX, Oak Harbor, WA, USA) valve fitted with a 10 µL injection loop.

### 2.5. HPLC-MS/MS

Ten microliters of extract analyzed with a MetaChem (Torrance, CA, USA) Inertsil C18 (150 mm length, 3 mm diameter, 5 μm diameter particle, 100 Å pore size) column. Chromatography was performed utilizing a ThermoSpectraPhysics (Thermo Scientific, San Jose, CA, USA) high performance liquid chromatography system consisting of an AS4000 autosampler coupled to a P2000 gradient pump. Elution of analyte was achieved with a 300 μL/min gradient flow of methanol and water (0.3% acetic acid was added to the mobile phase.) The solvent program used a 35–95% gradient over 25 min. The mass spectrometer was operated in positive mode electrospray ionization mode with an ionization voltage of 4.5 kV. The inlet capillary temperature was 255 °C, the inlet capillary voltage was 46 V and the tube lens offset was −5 V. The collision energy was optimized to 33%. Operation of the chromatography and mass spectrometry instrument and quantitation of the eluting 8-*O*-methylbostrycoidin was done utilizing ThermoFinnigan Xcalibur software (Version 1.4, Thermo Scientific, San Jose, CA, USA). Quantitative data was processed using Xcalibur QualBrowser (ThermoFinnigan) software. Uncertainties in mean determinations are expressed in terms of standard deviation.

Calibration curves were plotted using pure standards as well as maize matrix-assisted standards (acetonitrile extract). Maize matrix assisted standards were prepared as follows: Maize extract (considered blank for 8-*O*-methylbostrycoidin), which was obtained as described above, was spiked with 8-*O*-methylbostrycoidin dissolved at an appropriate concentration in acetonitrile.

### 2.6. Spike/Recovery Experiments and Method Validation

Spike recovery experiments were carried out in order to validate the method. Spiking solutions were prepared in acetonitrile and appropriate amounts added to cracked maize 1 h before extraction. Maize samples were spiked with 8-*O*-methylbostrycoidin by adding 250 µL aliquots (0.5, 5 and 50 µg/mL) of 8-*O*-methylbostrycoidin. Spiked samples were extracted with the same methods used for other samples. Spike/recovery samples were analyzed in triplicate on three successive days to evaluate method stability. Quantitations were based on integration of chromatographic peak area corresponding to the detection of characteristic fragment ions arising from the collisionally induced dissociation of the parent pseudomolecular ion [M + H]^+^ of the analyte. Analyte responses were compared to responses obtained from solubilized (acetonitrile) 8-*O*-methylbostrycoidin. 8-*O*-methylbostrycoidin was quantified using an external calibration curve, which was constructed by plotting the concentration against the signal area. 

### 2.7. Field Test

Maize line B73 was grown in field plots at the USDA, ARS, NCAUR (Peoria County, IL, USA) following guidelines of the Animal and Plant Health Inspection Service (APHIS) outlined in APHIS permit number P526-09-01825. The resulting maize ears were infected with the *F. verticillioides* by injection of 2 mL of a solution of 1 × 10^6^ *F. verticillioides* spores per mL water into the silk channel of each ear at 4 to 6 days after silk emergence [16]. Control ears were similarly injected with water. For each treatment 10 ears were inoculated. Upon maturity, ears were harvested and visually evaluated for extent of fungal infestation as described by Desjardins et al. [17]. The disease evaluation method provides a 0–7 scoring of the damage of the kernels of the ear where a higher score indicates greater damage and a lower score indicates lesser damage. The ears were shelled and the resulting kernels were ground with a Stein Model M2 Laboratory Mill (Steinlite Corporation, Atchison, KS, USA). Ground kernels for each ear were extracted for LC-MS/MS analysis.

## 3. Results

### 3.1. MS and MS/MS by Flow Injection

**Figure 2:**
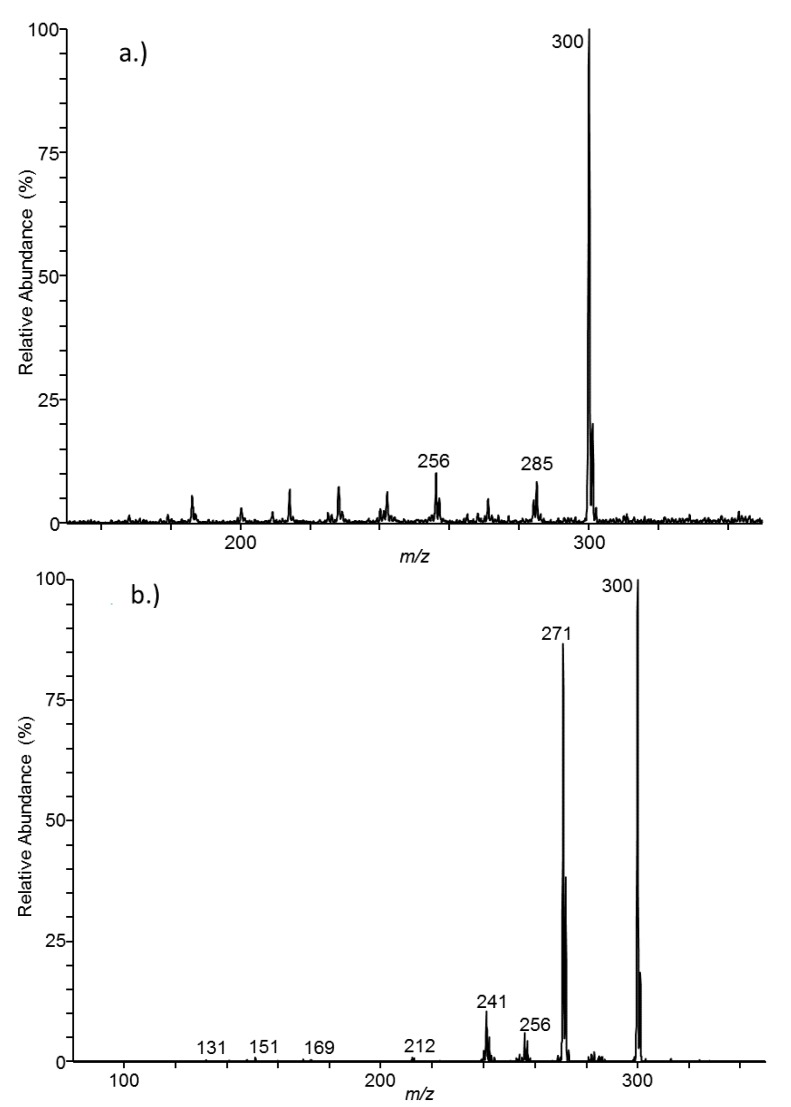
(**a**) MS and (**b**) MS/MS spectra for 8-*O*-methylbostrycoidin.

The ESI-MS spectrum for the flow injection of a solution of 8-*O*-methylbostrycoidin is shown in Figure 2a. Operating the ion-trap mass analyzer in the MS/MS mode has potential to increase the selectivity of the method, minimize background signal and improve method performance. To further assess 8-*O*-methylbostrycoidin mass spectrometric behavior, tandem mass spectra for the collisional induced dissociation of the [M + H]^+^ peak were acquired. When a certain threshold collision energy level was exceeded, the molecule broke up into several fragments. Features of the spectra did not change substantially despite use of higher collision energies. The resulting spectra were extremely simple. A spectrum for a CID-MS/MS experiment is shown in Figure 2b (33% collision energy). The instrument was operated to allow selection of a parent ion (*m*/*z* 300) and scanning of product ions. The dominant fragment ions at *m*/*z* 241, 256 and 271 are likely the result of facile losses of acetic acid, carbon dioxide and methanol. Other minor fragments are likely the result of extensive rearrangements. The molecular ion shows a great deal of stability despite use of relatively high collision energy. Lower mass fragments resulting from a more major decomposition of the parent ion would likely fall beneath the lower mass limit of the MS detector during the MS/MS experiment.

### 3.2. HPLC Coupled to MS

**Figure 3:**
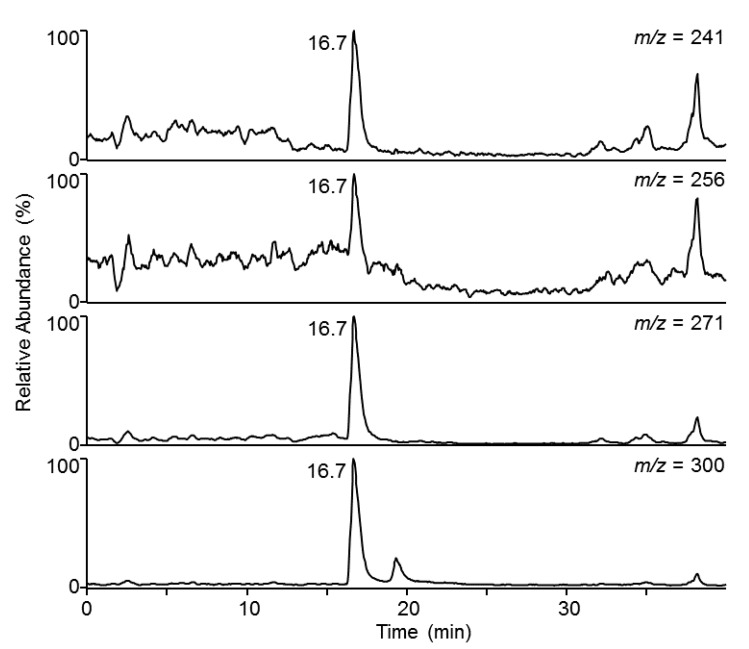
Chromatogram for elution of a 50 µg/mL solvent standard of 8-*O*-methylbostrycoidin.

Injections of 8-*O*-methylbostrycoidin (10 µL, 50 µg/mL) were made into reversed phase gradient flows of methanol and water on the HPLC column. Good chromatographic behavior was observed with 10 µL injections of concentrations up to 1000 µg/mL. Figure 3 shows a LC-MS/MS profile resulting from of a 50 µg/mL solvent standard injection using multiple reaction monitoring (MRM) reflecting detection of the three fragments and intact parent [M + H]^+^ ion. 

### 3.3. LC-MS/MS Quantitation

**Figure 4:**
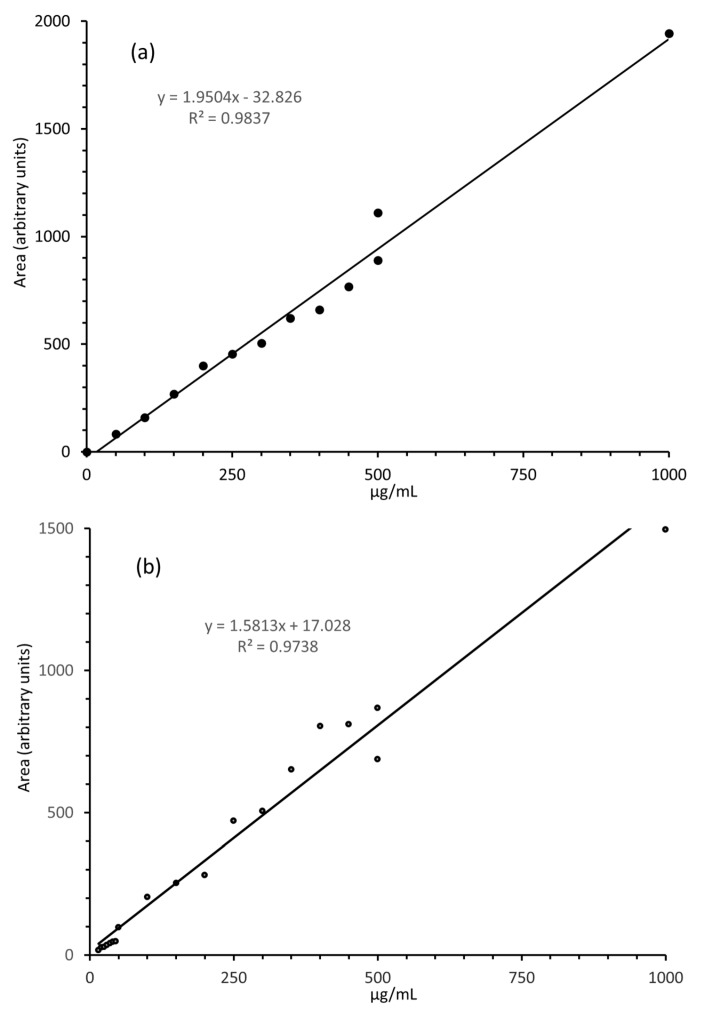
Calibration curves for 8-*O*-methylbostrycoidin (**a**) solvent and (**b**) matrix standards.

For quantitative analyses, abundant fragments from the [M + H]^+^ ion of the analyte, 8-*O*-methylbostrycoidin, were monitored in the MRM mode. Signals from the fragments were maximized by optimizing collision energy. To assess quantitative behavior of the LC-MS system, 10 µL injections over a wide range of 8-*O*-methylbostrycoidin concentrations were made. Response curves were obtained over a range of 8-*O*-methylbostrycoidin concentrations. Based on the observed response, it was determined that the monitoring of the *m*/*z* 271 fragment ion provided a good basis for quantitation of 8-*O*-methylbostrycoidin. Figure 4 shows an example plot of integrated response against concentration for the *m*/*z* 271 fragment ion from solvent and matrix standards. Good linearity, up to 1000 µg/mL, is observed. Based on the observation of the transition to *m*/*z* 271 ion, the absolute minimum detection limit (based on the signal-to-noise ratio of 3) was better than 3 ng/g from 8-*O*-methylbostrycoidin spiked into ground maize. Further, the limit of quantitation was 10 ng/g from 8-*O*-methylbostrycoidin spiked into ground maize.

### 3.4. Spike Recovery Studies

The goal of this work was to have a convenient method for determination of 8-*O*-methylbostrycoidin from maize substrate. To assess the ability of the LC-MS/MS technique to serve as the basis for such a method, spike-recovery experiments were conducted. A series of portions of cracked maize were spiked with 8-*O*-methylbostrycoidin (acetonitrile) in order to achieve levels corresponding to 0.0125, 0.125 and 1.25 µg/g 8-*O*-methylbostrycoidin. The samples were then extracted and processed according to the above described method. The analysis of the spiked samples (*n* = 9 for each spike level) yielded recoveries (72.8 ± 35.6, 0.0125 µg/g; 41.3 ± 17.5, 0.125 µg/g; 59.9 ± 32.3, 1.25 µg/g) of the 8-*O*-methylbostrycoidin upon quantitation utilizing the *m*/*z* 271 fragment ion.

### 3.5. Matrix Interference

Experiments to evaluate matrix effects were in conducted according to guidelines described by Matuszewski et al. [18]. LC-MS/MS areas of standards formulated in solvent (acetonitrile) were compared with those measured in a blank cracked maize extract spiked, after extraction, with the same analyte amounts. Tests were conducted on spiked maize extract samples (8-*O*-methylbostrycoidin concentration of 0.5 μg/mL). The level of 8-*O*-methylbostrycoidin in the maize extract used for the spiking experiments was assumed to be zero. The average of measured matrix effect (%) from solvent and extract samples run in pairs (*n* = 8) was 154 ± 12%. In the case of maize extract, the matrix actually provides an enhancement to the response of the LC-MS/MS detection. 

### 3.6. Determination of 8-O-methylbostrycoidin from Field Grown Maize

Field tests were conducted to evaluate the ability of *F. verticillioides* to produce 8-*O*-methylbostrycoidin in maize kernels under agriculturally relevant conditions. Injection of suspensions of spores from two different strains of *F. verticillioides* into developing maize ears resulted in relatively high levels of maize ear rot symptoms at harvest (Table 1). Injection of the different strains of *F. verticillioides* resulted in markedly different levels of 8-*O*-methylbostrycoidin in the resulting kernels (Table 1). Control injection of ears with water resulted in low levels of disease symptoms, as previous reported [17], and 8-*O*-methylbostrycoidin was not detected in kernels harvested from control-injected ears. 

## 4. Discussion

Currently, common methods for the evaluation of 8-*O*-methylbostrycoidin production by fungi are based upon UV absorbance. Potentially, such methods would be susceptible to interferences from extracted absorbing compounds produced by maize, as well as invading fungi. The method described here offers the potential of advantages in sensitivity and selectivity associated with LC-MS/MS. Our examination of the performance of the method provides us with an indication of its sensitivity, as well as its tolerance of matrix effects from the maize. The developed method allows the convenient determination of 8-*O*-methylbostrycoidin without a substantial effort for sample clean-up. As far as we are aware, this is the first report of production of 8-*O*-methylbostrycoidin in field-grown maize.

## Figures and Tables

**Table 1:** Results for field evaluation of disease level and 8-*O*-methylbostrycoidin production in maize ears upon inoculation with *F. verticillioides* isolates.

Strain	Disease Score ^1^	Level of 8-*O*-methylbostrycoidin (µg/g) ^1^
AMR 5	4.9 (1.9)	0.5 (0.9)
AMR 10	4.4 (1.6)	2.6 (4.8)
Control inoculation	1.3 (0.9)	0 (–)

^1^ Uncertainties in determinations in terms of standard deviations are indicated in parentheses. Each maize ear was considered a repetition of the inoculation experiment *n* = 10. No standard deviation was calculated for the levels of the analyte detected in the control inoculation kernels. This is indicated by the (–).
